# *In silico* Identification and Validation of a Linear and Naturally Immunogenic B-Cell Epitope of the *Plasmodium vivax* Malaria Vaccine Candidate Merozoite Surface Protein-9

**DOI:** 10.1371/journal.pone.0146951

**Published:** 2016-01-20

**Authors:** Rodrigo Nunes Rodrigues-da-Silva, João Hermínio Martins da Silva, Balwan Singh, Jianlin Jiang, Esmeralda V. S. Meyer, Fátima Santos, Dalma Maria Banic, Alberto Moreno, Mary R. Galinski, Joseli Oliveira-Ferreira, Josué da Costa Lima-Junior

**Affiliations:** 1 Laboratório de Imunoparasitologia, Instituto Oswaldo Cruz, Fundação Oswaldo Cruz (FIOCRUZ), Rio de Janeiro, RJ, Brazil; 2 Computational Modeling Group—FIOCRUZ-CE, Fortaleza, Brazil; 3 Emory Vaccine Center, Yerkes National Primate Research Center, Emory University, Atlanta, GA, United States of America; 4 Environmental Health and Safety Office, Emory University, Atlanta, GA, United States of America; 5 National Health Foundation, Department of Entomology, Central Laboratory, Porto Velho, RO, Brazil; 6 Laboratory of Simulids and Onchocerciasis "Malaria and Onchocerciasis Research", Oswaldo Cruz Institute, Oswaldo Cruz Foundation, Rio de Janeiro, Brazil; 7 Division of Infectious Diseases, Department of Medicine, Emory University School of Medicine, Emory University, Atlanta, GA, United States of America; Universidade Federal de Minas Gerais, BRAZIL

## Abstract

Synthetic peptide vaccines provide the advantages of safety, stability and low cost. The success of this approach is highly dependent on efficient epitope identification and synthetic strategies for efficacious delivery. In malaria, the Merozoite Surface Protein-9 of *Plasmodium vivax* (PvMSP9) has been considered a vaccine candidate based on the evidence that specific antibodies were able to inhibit merozoite invasion and recombinant proteins were highly immunogenic in mice and humans. However the identities of linear B-cell epitopes within PvMSP9 as targets of functional antibodies remain undefined. We used several publicly-available algorithms for *in silico* analyses and prediction of relevant B cell epitopes within PMSP9. We show that the tandem repeat sequence EAAPENAEPVHENA (PvMSP9_E795-A808_) present at the C-terminal region is a promising target for antibodies, given its high combined score to be a linear epitope and located in a putative intrinsically unstructured region of the native protein. To confirm the predictive value of the computational approach, plasma samples from 545 naturally exposed individuals were screened for IgG reactivity against the recombinant PvMSP9-RIRII_729-972_ and a synthetic peptide representing the predicted B cell epitope PvMSP9_E795-A808_. 316 individuals (58%) were responders to the full repetitive region PvMSP9-RIRII, of which 177 (56%) also presented total IgG reactivity against the synthetic peptide, confirming it validity as a B cell epitope. The reactivity indexes of anti-PvMSP9-RIRII and anti-PvMSP9_E795-A808_ antibodies were correlated. Interestingly, a potential role in the acquisition of protective immunity was associated with the linear epitope, since the IgG1 subclass against PvMSP9_E795-A808_ was the prevalent subclass and this directly correlated with time elapsed since the last malaria episode; however this was not observed in the antibody responses against the full PvMSP9-RIRII. In conclusion, our findings identified and experimentally confirmed the potential of PvMSP9_E795-A808_ as an immunogenic linear B cell epitope within the *P*. *vivax* malaria vaccine candidate PvMSP9 and support its inclusion in future subunit vaccines.

## Introduction

Despite global investments in the control and elimination of malaria, the disease remains a major public health burden worldwide. According to the World Health Organization (WHO), more than 3 billion people are still at risk of infection, with an estimated 197 million of cases and 584 thousand deaths [1]. Among the species that infect humans *Plasmodium falciparum* and *P*. *vivax* are considered the two most important malaria parasites. Although *P*. *falciparum* is responsible for the major number of cases and deaths, especially in children, *P*. *vivax* is the most prevalent species outside the African continent [1]. Aside from the enormous socioeconomic impact caused by *P*. *vivax* prevalence [2], an increased number of publications reporting severe disease [3–8] and the emergence of strains resistant to chloroquine [9–11] and primaquine [12–14], make the development of a safe and affordable vaccine an important component in *P*. *vivax* control strategies. Although the epidemiological importance of *P*. *vivax* malaria worldwide is evident, the research on potential *P*. *vivax* vaccine candidates lags behind that on *P*. *falciparum*. Currently, there are only four *P*. *vivax* vaccine candidates or components in advanced preclinical studies and only one in clinical development, while 34 *P*. *falciparum* candidates are as listed in the WHO’s Malaria Vaccine Rainbow Tables [15]. These data show the continued global commitment to control and eliminate malaria with strategies that include vaccination, and highlight the specific need for identifying and testing additional vaccine candidates against *P*. *vivax*.

Recent advances in adjuvant composition, delivery systems and the design of subunit vaccine constructs, support the use of synthetic peptides containing B and T-cell epitopes as a vaccine platform against malaria. Moreover, synthetic peptide vaccines have several advantages for clinical development, such as their stability in the absence of proteases, the lack of contamination with biological agents, the fast production with good inter-batch reproducibility, and the facility to be produced using solid phase peptide synthesis technologies that do not require skilled operators [16]. In *P*. *vivax* vaccine studies, long synthetic peptide (LSP) vaccines have been shown to be immunogenic in New World monkeys of the genus *Aotus* [16] and they were reported to be safe and immunogenic in phase I clinical trials [17]. The LSP approach allows the combination of different epitopes of different vaccine targets, a strategy that has had success in murine malaria models [18]. The identification of antigens that induce protective responses and confirmation of their immunogenic potential are critical for effective vaccine development using synthetic platforms.

**Fig 1:**
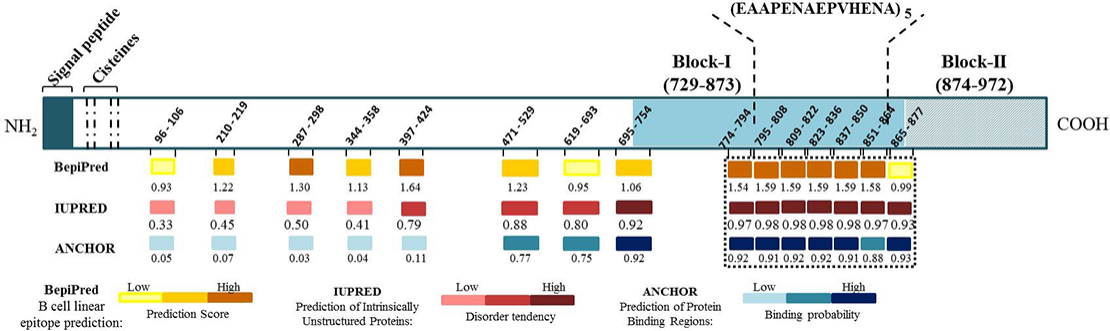
Schematic diagram of PvMSP9 and the predictions scores for linear B cell epitopes, intrinsically unstructured/disordered regions and protein-protein interaction regions. The region corresponding to the amino acid residues 795–808 of PvMSP9 was selected for the synthesis of a soluble peptide based on the best combination of prediction scores using BepiPred, IUPRED and ANCHOR algorithms. Yellow heat bars represent B-cell epitopes, red heat bars represent predicted unordered regions and blue heat bars represent prediction of binding regions. The prediction scores represents the average of scores for all amino acids within the region with prediction values above the cut-offs chosen for significance. The bar color intensities are proportional to the prediction scores.

Invasion of erythrocytes is a critical step in the *Plasmodium* life cycle that is associated with clinical manifestations and complications. Vaccines targeting this stage are intended to reduce morbidity and mortality [19]. Erythrocytic vaccine strategies aim to disrupt the interaction between *Plasmodium* merozoite proteins and erythrocyte surface ligands by eliciting neutralizing antibodies [20, 21], an approach strongly supported by studies with asexual blood-stage antigens in animal models [22] and immune recognition of these antigens by exposed individuals in malaria-endemic areas [23–27]. In this scenario, Merozoite Surface Proteins (MSP) are a promising set of proteins, since they are expressed during schizogony and become associated with the surface of merozoites in the course of schizont development [28]. Moreover, based on their repeated exposure to the host immune system, several MSPs were described and their immunological properties were investigated [29–31]. Among these proteins, PvMSP9 has gained attention as a potential vaccine candidate. The MSP9 was initially identify in *Plasmodium falciparum* as a 101 kDa Acidic-Basic Repetitive Antigen (ABRA/PfMSP9), and then orthologous genes were identified in other *Plasmodium* species [28, 32, 33]. The phylogeny of MSP9 shows that *P*. *vivax* and species of *Plasmodium* that infect non-human primates are closely related [34]. Structurally, *P*.*vivax* MSP9 was described as a hydrophilic protein with a putative 20 amino acid signal peptide, a cluster of four cysteines, a long non-repetitive conserved N-terminal domain and a C-terminal region containing blocks of species-specific tandem repeats [28, 35] (Fig 1). Previous studies have demonstrated that the N-terminal region was immunogenic in mice [36], and naturally acquired immune responses have been described in adults [26] and children [31]. The immunogenic N-terminal region contains five promiscuous T-cell epitopes (pE, pJ, pK, pH and pL), which interact with a broad range of HLA class II molecules [36, 37]. Concerning the C-terminal region, naturally acquired immune responses of adults living in malaria endemic areas, confirmed the presence of highly antigenic blocks of tandem repeats (RI and RII). Anti-PvMSP9-RIRII antibodies are directly correlated to malaria exposure [26, 35]. These observations, allied to the ability of a PvMSP9 monoclonal antibody to inhibit *P*. *vivax* merozoite invasion into erythrocytes [28], suggest that PvMSP9 contains potential B-cell epitopes that could be used in the design of a multi-target vaccine candidate against *P*. *vivax*.

Pertinent to this context, most protein epitopes are thought to be discontinuous, composed of different parts of the polypeptide chain that are brought into spatial proximity by the folding of the protein. However, for approximately 10% of the epitopes, the corresponding antibodies are cross-reactive with a linear peptide fragment of the epitope [38], those linear or continuous epitopes are comprised of a single stretch of the polypeptide chain. In the post-genomic era, reverse vaccinology approaches have gained attention for the rational selection of antigens and identification of key immunological epitopes [39]. Consequently, the efficient prediction and confirmation of immunogenic linear epitopes also represents a promising strategy to develop safe, viable and cost-effective vaccines. The aim of the present study was to identify an antigenic B-cell linear epitope within PvMSP9 and confirm its immunogenicity by using a synthetic peptide representing the predicted epitope for seroepidemiological studies. Our data add further support for studies of vaccines based on linear synthetic-peptides and epitope mapping strategies to characterize *Plasmodium vivax* antigens.

## Material and Methods

### Sequence Data

To predict possible antigenic properties and the 3-Dimensional (3D) structure of PvMSP9 (PlasmoDB ID: PVX_124060) using bioinformatic tools, the entire sequence of PvMSP9 (Belem strain, Accession Number AAL78897.1) was downloaded from the NCBI website (www.ncbi.nlm.nih.gov/protein) and used for analyses.

### B Cell Epitope Prediction

The prediction of linear B-cell epitopes was carried out using the program BepiPred [38]. This software takes a single sequence in FASTA format input and each amino acid receives a prediction score based on Hidden Markov Model profiles of known antigens and incorporates propensity scale methods based on hydrophilicity and secondary structure prediction. For each input sequence the server outputs a prediction score. The positions of the linear B-cell epitopes are predicted to be located at the residues with the highest scores. In order to consider a given region as a valid linear B cell epitope for PvMSP9, the cut-off value of 0.9 was used to warrant a high specificity (0.91) and low predicted sensitivity (0.25). Therefore, the epitope score represents the average of the scores of least nine consecutive amino acids above the cut-off, and the sequences with higher mean values were chosen as potential linear epitopes.

### Prediction of Intrinsically Unstructured/Disordered Regions (IURs) and Potential Binding Regions in PvMSP9

The prediction of intrinsically unstructured/disordered regions (IURs) was carried out using the IUPred algorithm [40]. IUPred takes a single sequence in FASTA format as input and predicts the potential IURs. The final output is an individual score for each amino acid that ranges from 0 (completely ordered) to 1 (completely unordered). IURs were then predicted as a region spanning at least 9 contiguous amino acids with individual IUPred prediction score for each amino acid >0.5. Additionally, the ANCHOR tool was used to predict possible regions involved in protein-protein interactions in the complete PvMSP9 sequence. This approach relies on the pairwise energy estimation approach and seeks to identify segments that reside in disordered regions, cannot form enough favorable intra-chain interactions to fold on their own and are likely to gain stabilizing energy by interacting with a globular protein partner [41]. The basic output of this prediction method is a probability score, indicating the likelihood of the residue to be part of a disordered binding region along each position in the sequence. Regions that have a score >0.5 and pass the filtering criteria are predicted as disordered binding regions.

### 3D Model and Electrostatic Analysis

The 3D structure of MSP9 was predicted using the Robetta algorithm [42]. The amino acid sequence was retrieved from NCBI under accession code AAL78897.1. The Robetta is an automated algorithm for predictions of the 3D structure of proteins through *ab initio* and comparative modeling. The first step is the searching for structural homologs using BLAST [43] or PSI-BLAST [44]. In the protein sequence, the target primary structure is broken down into separated domains, or independently folding units of proteins, by comparing the sequence to structural families in the Pfam database [45]. Domains with homolog structures follow a template-based modeling protocol. The final five structures are selected by taking the lowest energy models as determined by the Rosetta energy function. The electrostatic surface was calculated with the Adaptive Poisson-Boltzmann Solver (APBS) software [46] integrated with Pymol [47]. The APBS software solves the Poisson-Boltzmann equation in order to describe electrostatic interactions between solute in aqueous solution. Continuous electrostatic plays a very important role in determining ligand-protein and protein-protein binding kinetics.

### Molecular Dynamics Simulations

The GROMACS 4.6.5 package [48] was used to perform the minimization and dynamics of the PvMSP9 protein under explicit solvent. Dynamics simulations were run with the GROMOS96 53a6 force field [49] and the SPC water model [50]. The MSP9 model was energy minimized then the system was gradually heated from 0 to 300 K over 3ns using the NVT ensemble with the Berendsen thermostat. A total of 10ns was performed.

### Peptide Synthesis

The consensual analysis of the *in silico* prediction tools, indicate the peptide sequence corresponding to residues E_795_–A_808_ as a relevant epitope within PvMSP9. Therefore, the sequence EAAPENAEPVHENA was synthesized by fluorenylmethoxycarbonyl (F-moc) solid-phase chemistry [51] (GenOne Biotechnologies, Brazil). Analytical chromatography of the peptide demonstrated a purity of >95% and mass spectrometric analysis also indicated an estimated mass of 1477.50 Da, corresponding to the mass of the peptide.

### Protein Expression

PvMSP9 recombinant proteins representing the C-terminal region containing the second block of repeats (PvMSP9-RII; aa874-972) [35] and containg Blocks I and II of tandem repeats (PvMSP9-RIRII; aa 729–972) [26] were initially amplified from *P*. *vivax* (Belem strain), expressed as GST fusion proteins and purified as previously described. The SDS-PAGE of PvMSP9-RIRII recombinant protein used in ELISA assays is shown in S1 Fig.

### Samples and Survey

Plasma samples were examined from a cross-sectional cohort study involving 545 individuals from communities in the malaria endemic region of Rondônia state, Brazil, where over the last seven years, *P*. *vivax* malaria accounts for more than 80% of all malaria cases [52]. The individuals in the study population have been described elsewhere [26]. Briefly, they consist of rain forest natives as well as migrants from several non-endemic areas of Brazil who have resided in the region for 5 years or more. Additionally, samples from 24 individuals from non-endemic regions of Brazil, who never resided in malaria endemic areas and with no history of malaria, comprised a control group. A study survey included questions related to demographics, time of residence in the endemic area, personal histories of malaria and personal knowledge of malaria. The enrollment exclusion criteria were as follows: age <10 years old, pregnancy, breast-feeding, anti-malarial drug use, mental disorders and status as member of an indigenous population. Written informed consent was obtained from all adult donors or from parents of donors in the case of minors. The study was reviewed and approved by the Oswaldo Cruz Foundation Ethical Committee and the National Ethical Committee of Brazil.

### Detection of Specific Antibodies against the Recombinant PvMSP9-RIRII and the Predicted B Cell Epitope EAAPENAEPVHENA (PvMSP9_E795-A808_)

Plasma samples from study participants were screened for the presence of naturally acquired antibodies against the PvMSP9-RIRII recombinant protein and PvMSP9_E795-A808_ synthetic peptide by enzyme-linked immunosorbent assay (ELISA). Briefly, MaxiSorp 96-well plates (Nunc, Rochester, NY) were coated with 5 μg/mL of peptide or 2 μg/mL of recombinant protein. After overnight incubation at 4°C, plates were washed with PBS and blocked with PBS-Tween containing 5% non-fat dry milk (PBS-Tween- M) for 2h at 37°C. Individual plasma samples diluted 1:100 on PBS-Tween-M were added in duplicate wells and the plates incubated at 37°C for 2h. After three washes with PBS-Tween, bound antibodies were detected with peroxidase-conjugated goat anti-human IgG (Sigma St. Louis, MO) followed by o-phenylediamine and hydrogen peroxide. The absorbance was read at 492nm using an ELISA reader (Spectramax 250, Molecular Devices, Sunnyvale, CA) and specific reactivity was obtained by subtraction of the averaged OD value due to GST alone from the averaged OD value of the same plasma for the recombinant protein. The results for total IgG were expressed as reactivity indexes (RI) that were calculated by dividing the mean optical density (OD) of tested samples by the mean ODs plus 3 standard deviations (SD) of 24 non-exposed controls. Subjects were considered IgG responders to a particular antigen if the RI was higher than 1. The total IgG responders were also tested for IgG subclasses using the following peroxidase-conjugated monoclonal mouse anti-human antibodies: clones HP-6001 for IgG1, HP-6002 for IgG2, HP-6050 for IgG3 and HP-6023 for IgG4 (Sigma), as described before. As the cut-off for positivity, subclass-specific prevalence for each antigen was determined using OD values above 3 SD mean OD of 24 non-exposed controls.

### Statistical Analysis

All statistics analyzes were carried out using Prism 5.0 for Windows (GraphPad Software, Inc.). The one-sample Kolmogorov-Smirnoff test was used to determine whether a variable was normally distributed. The Wilcoxon matched pairs test was used to compare reactivity indexes of synthetic peptides and recombinant protein (PvMSP9-RIRII) and the optical density (OD) against PvMSP9-RIRII on absorption ELISA. Differences in proportions of the RI of IgG subclasses were evaluated by chi-square test (χ2) and associations between antibody responses and epidemiological data were determined by the Fisher’s exact test or the Spearman rank test when appropriated. A two-sided P value < 0.05 was considered significant.

## Results

### *In silico* Analysis of PvMSP9 and Identification of PvMSP9_E795-A808_ as a Potential B Cell Epitope

To detect potential linear B-cell epitopes with intrinsically unstructured/disordered regions and possible components of binding regions in the protein, the full sequence of PvMSP9 was analyzed using the BepiPred, IUPRED and ANCHOR algorithms, respectively. As shown in Fig 1, nine high scored potential linear epitopes with at least nine amino acids were identified on the entire protein sequence. The prediction scores ranged from 0.93 to 1.64. However, a long fragment of 104 amino acids (E_774_ –H_877_; prediction score mean of 1.5) was identified as a main epitope within the known naturally immunogenic C terminal region. E_774_-H_877_ was further characterized and seven linear epitopes were predicted: the first sequence 774–794 with a BepiPred score of 1.54, five uninterrupted tandem repeats of the sequence EAAPENAEPVHENA (E_795_-A_808_; E_809_-A_822_; E_823_-A_836;_ E_837_-A_850_; E_851_-A_864_) and the last predicted sequence 865–877 with a BepiPred score of 0.88. The five tandem repeats represented 29% of all PvMSP9-RIRII amino acid residues and also presented the highest epitope prediction score (mean = 1.59) within the repetitive C-terminal region of PvMSP9 and the second highest prediction score of the full sequence. In relation to the probability of being a binding site and having the presence of intrinsically unstructured regions, the N terminal region presented lower scores (0.06 and 0.49, respectively) and the central region had intermediate scores (0.61 and 0.86). Interestingly, the epitopes located in the C-terminal region (E_774_-H_877_) presented the highest disorder tendency score of the protein sequence (0.97) and presented a high probability of being a binding region (0.92). The consensual analysis of prediction scores indicated that the tandem sequence of repeats contained an important epitope within the PvMSP9 region predicted to be involved in protein-protein interactions. Therefore, the putative predicted sequence EAAPENAEPVHENA was designated PvMSP9_E795-A808_ and selected for further characterization of its potential as a B cell epitope using a synthetic peptide and naturally acquired antibodies.

### Molecular Modeling of the PvMSP9 RIRII Domain

**Fig 2:**
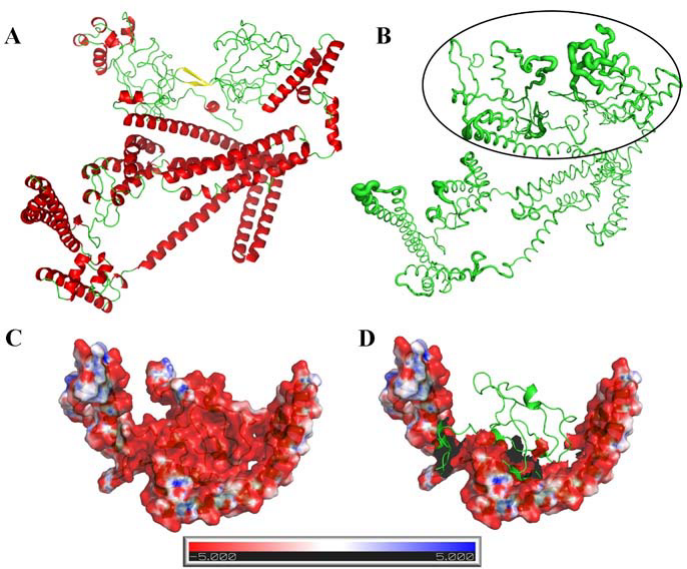
Three-dimensional structure prediction of the PvMSP9 RIRII domain. (A) 3D model of the PvMSP9 domain constructed using the Robetta algorithm. Red structures depict alpha helices, while the disordered region is represented in green. A small beta sheet was found between residues 850 and 858. (B) B-factor as calculated by GROMACS after 10ns simulation. The thicker segments represent the most flexible regions, while the thinnest represent the most rigid. (C) Electrostatic surface of the disordered region, showing a predominantly negative segment in red. (D) Same region show in B, without the electrostatic surface, showing the region 793–866 highlighted in green. The color scale was set from 5 kT/e (red) to 5 kT/e (blue), as calculated by APBS.

The predicted PvMSP9 RIRII structure is composed of 53% alpha helices, less than 1% beta sheets and 45% turns and coils, as measured by Stride [53]. The disordered regions are located in the RIRII domain, represented by green loops (Fig 2A). The PvMSP9 surface is highly charged, with a high number of Asp and Glu residues (Fig 2C and 2D). The region encompassing the PvMSP9-RIRII predicted region seems more flexible than the rest of the structure, shown by the calculated B-factors (Fig 2B). The B-factors of protein crystal structures reflect the overall fluctuation of atoms about their average positions and are capable of providing important information about protein dynamics. This result confirms the prediction of the disordered region defined by BepiPred. Also, it is possible to notice the relative exposition to the solvent and consequently, other receptors or ligands.

### Epidemiological Profile of the Studied Individuals

The epidemiological characteristics of the studied population are summarized on Table 1. The participants of the study were mostly composed of men (Χ^2^: 14.98, p = 0.0001), with ages ranging from 10 to 85 years old. All enrolled individuals were residing in areas where malaria transmission occurs and were considered naturally exposed to *P*. *vivax* infections. As a consequence, 85.7% of participants reported previous malaria episodes and 73.4% reported previous infections with *P*. *vivax*. At the moment of diagnosis, 11.4% of the population was infected and received the appropriate treatment according to the Brazilian guidelines (Table 1).

**Table 1:** Summary of the epidemiological data of naturally exposed individuals enrolled in the study.

	Median (IQ)	Frequency (N)
**Gender**		
Male	-	55.9% (305)
Female	-	44.1% (240)
**Malaria exposure**		
Age	33 (21–49)	-
Years of residence on endemic area	23 (16–37)	-
Number of past malaria infections	5 (2–10)	-
Months since the last malaria infection	1 (0–12)	-
**Previous malaria species contracted**		
*Plasmodium vivax*	-	15.8% (86)
*Plasmodium falciparum*	-	10.8% (59)
Both species	-	57.6% (314)
Never infected / Not reported	-	15.8% (86)
**Diagnosis**		
*Plasmodium vivax*	-	6.8% (37)
*Plasmodium falciparum*	-	4.6% (25)
*P*. *falciparum + P*. *vivax*	-	0% (0)
Not infected	-	89% (483)

### Characterization of PvMSP9_E795-A808_ as a Naturally Immunogenic B-Cell Linear Epitope within the Immunodominant Region of PvMSP9

**Fig 3:**
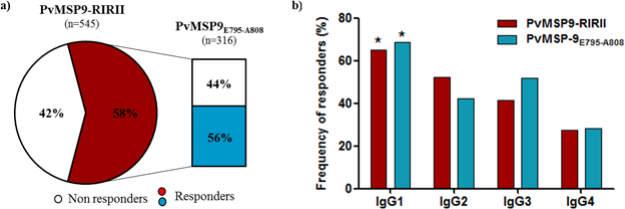
Frequency of total IgG and IgG subclasses responders to PvMSP9-RIRII and to PvMSP9_E795-A808_. (A) Frequency of total IgG responders to PvMSP9-RIRII (red pie slice) and PvMSP9_E795-A808_ (blue bar). (B) Frequency of IgG subclasses responders to PvMSP9-RIRII and PvMSP9_E795-A808_ presented no statistically significant difference. (*) Indicates that the difference was significant (p < 0.05) for a comparison between a particular IgG subclass over the others IgG subclasses for the same antigen by chi-square test.

**Fig 4:**
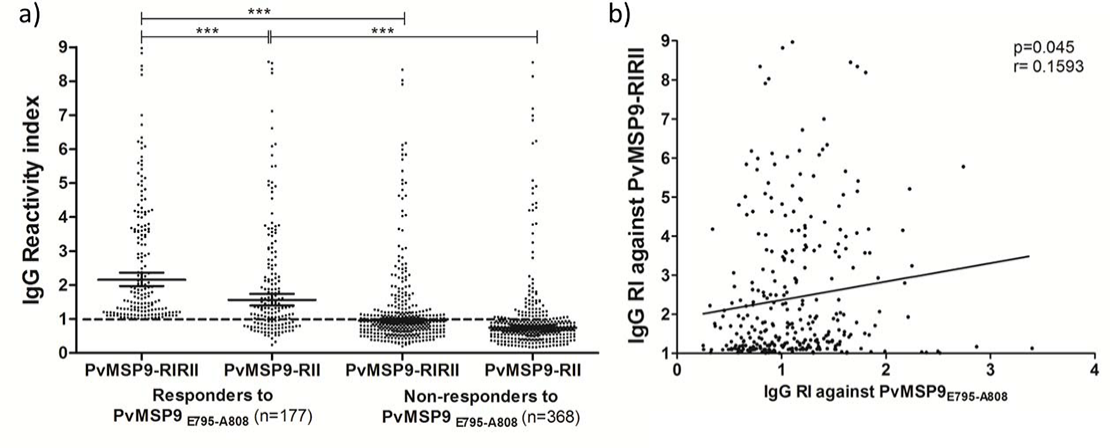
IgG reactivity index to PvMSP9-RIRII and PvMSP9_E795-A808_. (A) Comparison of IgG reactivity index against PvMSP9-RIRII and PvMSP9-RII among responders and non-responders to PvMSP9_E795-A808_. The lines indicate geometric means with 95% of confidence interval. The Mann Whitney test was used to compare medians of IgG reactivity indexes against recombinant proteins on responders and non-responders to synthetic peptide. Significant differences were indicated by *. (*) p<0.05; (**) p<0.001; *** p<0.0001 (B) Correlation between IgG reactivity indexes against PvMSP9_E795-A808_ and against PvMSP9-RIRII. The correlation was assessed by Spearman’s rank test.

To test if the PvMSP9-RIRII protein region is a target for naturally acquired antibodies, we assessed the IgG reactivity profile against the recombinant protein representing the two blocks of repeats from plasma samples collected from 545 individuals living in endemic areas of a western amazon region of Brazil. We observed that 58% of the studied population represented antibody responders against the recombinant PvMSP9-RIRII protein. Among the responders, the reactivity index ranged from 1.1 to 9.0 (mean = 2.4 ± 1.7), which reflected a wide spectrum in the magnitude of the naturally acquired IgG response and also confirmed the two blocks of repeats as an immunogenic region of PvMSP9. To test if the predicted PvMSP9_E795-A808_ sequence contains a valid B-cell epitope, we characterized the antigenicity of a synthetic peptide representing this sequence. The overall frequency of responders to the peptide was 32.5%, however among the 316 antibody responder individuals to PvMSP9-RIRII, 56% presented specific IgG antibody response against the PvMSP9_E795-A808_ synthetic peptide (Fig 3A). The magnitude of the anti-PvMSP9_E795-A808_ specific IgG response varied with RI values ranged from 1.1 to 3.4 (mean = 1.4 ± 0.4) in responders to PvMSP9_E795-A808_. Additionally, the IgG subclass profile against the synthetic PvMSP9_E795-A808_ was characterized with a significantly higher frequency of IgG1 responders (68.6%) over IgG2 (42.2%; χ2 = 13.41 p<0.0003); IgG3 (52%, χ2 = 5.24 p<0.0221) and IgG4 (28.2%, χ2 = 25.86 p<0.00001). A similar profile of IgG subclasses against PvMSP9-RIRII was observed with no significant differences between frequencies of IgG subclasses against the synthetic and recombinant PvMSP9 derived antigens (Fig 3B). After validating that the tandem repeat region within PvMSP9-RIRII is a linear B-cell epitope, we further evaluated the importance of PvMSP9_E795-A808_ by comparing the fine specificity of the naturally acquired antibody responses. Individuals with antibody responses to PvMSP9_E795-A808_ had higher IgG levels against PvMSP9-RIRII in comparison to non-responder individuals (p<0.0001; Fig 4A). Moreover, as shown in Fig 4A, we also observed that the significantly higher reactivity indexes against PvMSP9-RIRII when compared to PvMSP9-RII recombinant protein is present only among the responders to PvMSP9_E795-A808_, while this significant difference was not observed when the RIs against the repetitive regions was compared among non-responders to peptide. Lastly, as shown in Fig 4B, a weak direct correlation between the RI of IgG against PvMSP9_E795-A808_ and PvMSP9-RIRII (p = 0.0045; r = 0.1593) was also observed.

### Association between Epidemiological Variables and Antibody Responses to PvMSP9_E795-A808_

To assess whether epidemiological factors influence the naturally acquired immune response against PvMSP9_E795-A808_, different variables of the studied population were studied for correlation with the reactivity indexes of total IgG. Our data indicate that the reactivity index IgG specific against PvMSP9_E795-A808_ was not correlated with number of previous malaria infections (PMI; p = 0.733), time of residence in endemic area (TREA; p = 0.171) or time since the last malaria episode (TLM; p = 0.109). However, among responders to PvMSP9-RIRII, both responders and non-responders to PvMSP9_E795-A808_ presented significantly higher medians of time of residence in endemic areas (p<0.0001 and p = 0.0001 respectively) and time since the last malaria episode (p = 0.0225 and p = 0.0278 respectively) than non-responders to PvMSP9-RIRII (Table 2). In addition, a direct correlation between RIs against PvMS9-RIRII and time of residence in endemic areas was observed in both groups, responders (r = 0.3619; p<0.0001) and non-responders (r = 0.2560; p<0.002) to the peptide. Interestingly, the responders to PvMSP9_E795-A808,_ presented a RI of IgG against PvMSP9-RIRII that directly correlated with the time since the last malaria episode (p = 0.022, r = 0.177) and inversely correlated with the number of infections in the last six months (p = 0.0174, r = -0.178), while the non- responders presented no correlation with these parameters. Lastly, in relation to the IgG subclasses against PvMSP9_E795-A808_, PMI and TREA did not correlate with the RI for all tested subclasses. However, an IgG1 biased response directed to the epitope was associated with a protection parameter, since TLM was correlated with IgG1 reactivity indexes against PvMSP9_E795-A808_ (r = 0.2644, p<0.0362) but not against PvMSP9-RIRII (r = -0.0870, p- = 0.241).

**Table 2:** Epidemiological parameters grouped in different IgG reactivity profiles.

Epidemiological parameters	PvMSP9_E795-A808_ (+) PvMSP9-RIRII (+) (n = 177)	PvMSP9_E795-A808_ (-) PvMSP9-RIRII (+) (n = 139)	PvMSP9_E795-A808_ (-) PvMSP9-RIRII (-) (n = 230)
Years of residence in endemic area (Median-IQ)	29 (20–45) ^###^	26.5 (17–37) ^***^	19 (15–28) ^***^^;^ ^###^
Months elapsed since the last malaria episode (Median-IQ)	24 (2–72) ^#^	16 (2–72) ^*^	12 (2–48) ^*^^;^ ^#^
Number of previous malaria infections (Median-IQ)	5 (2–10)	5 (2–10)	5 (2–10)
Infections in the current year (Mean+SD)	0.44 ± 0.98	0.64 ± 1.46	0.68 ± 1.34
Infected by *P*. *vivax* at the moment of collection (N—%)	16 (9%)	6 (4%)	25 (11%)

Differences in proportions of number of individuals infected by *P*. *vivax* at the moment of collection were evaluated by the chi-square test (χ2) and comparisons of epidemiological parameters were made using the Mann Whitney test.

Significant differences between responders and non-responders to both antigens were indicated in the table by

#: p<0.05

##: p<0.001

###: p<0.0001.

Significant differences between responders to PvMSP9-RIRII only and non-responders were indicated in the table by

*: p<0.05

**: p<0.001

***: p<0.0001.

Statistical differences on epidemiological parameters were not observed between responders and non-responders to PvMSP9_E795-A808_.

## Discussion

The development of a safe, efficacious and inexpensive vaccine against *P*. *vivax* remains a challenge for the scientific community. Despite a considerable number of antigens that have been described as vaccine candidates, the conventional vaccinology strategies applied are especially difficult when dealing with non-cultivable microorganisms, as *P*. *vivax*. With the concomitant advent of whole-genome sequencing and advances in bioinformatics, the vaccinology field has been radically changed in the last few decades, providing the opportunity for description of novel antigens and improvement of the already known candidates. The vaccine constructs based on synthetic peptides represent one of these well succeeded reemerging strategies [54, 55], but it is strongly dependent of an efficient epitope selection. In this study we describe the identification of a B-cell linear epitope (PvMSP9_E795-A808_) within the *P*.*vivax* MSP9 using bioinformatics tools applied to reverse vaccinology. Using conventional vaccinology approaches, we validated PvMSP9_E795-A808_ as a target of antibodies by conducting a seroepidemiological assessment using a cohort of individuals naturally exposed to *P*. *vivax* in malaria endemic regions of western Brazil. Our results support further development of this epitope as a possible subunit in a multi-target synthetic vaccine against *P*. *vivax*.

Firstly, we screened the full sequence of PvMSP9 using the BepiPred algorithm. The selection of this epitope prediction algorithm was based on the fact that it is the heavily cited and widely used tool for *In silico* analyses of linear B-cell epitopes [38]. In *P*. *vivax* vaccine research this approach was recently used to map potential epitopes in well-known vaccine candidate PvMSP-1 [56] and also to map and validate a highly immunogenic linear epitope in PvAMA-1 [57]. The *in silico* mapping of PvMSP9 B cell epitopes by BepiPred indicated nine potential regions in the full protein sequence. In comparison to other *P*. *vivax* vaccine candidates, PvMSP9 presented a comparable number of epitopes predicted and higher predicted mean scores. Interestingly, the repetitive region of PvMSP9 predicted here is located within a long fragment in the C-terminal region previously identified as target of naturally acquired immune responses [26, 58], suggesting that the long sequence of 104 amino acids could be a main target of antibodies directed to PvMSP9_E795-A808_. Based on the evidence that the fragment predicted as a B-cell epitope contains five uninterrupted tandem repeats of the sequence: EAAPENAEPVHENA (_E795-A808; E809-A822; E823-A836; E837-A850; E851-A864_), each tandem sequence was analyzed as an individual epitope. The epitope based on the predicted tandem sequence of PvMSP9 is located in a species-specific region [28] with limited polymorphism [34, 35]. These findings supported the selection of this region as a main target for a linear B-cell epitope selection. In addition, allied to the high prediction score as a linear epitope, the sequence EAAPENAEPVHENA had the highest probability to be present in an intrinsically unstructured region of the PvMSP9 sequence. Interestingly, several vaccine candidates that have been extensively studied in *P*. *falciparum*, were later reported to have unstructured regions, some of which serve as targets of protective immunity. For example, MSP-2 was shown to be largely unstructured [59, 60], MSP-3 and Glutamate-rich protein (GLURP) presented long unstructured regions [61] and even Apical Membrane Antigen -1 (AMA-1), though generally known as a well-structured molecule [62], contains disordered N-and C-terminal regions [63]. In *P*. *vivax*, a recently described linear epitope within domain II of AMA-1, which was targeted for naturally acquired antibodies, is also located in an IUR. In this context, since many disordered proteins are organized via binding to a structured partner to gain stabilizing energy and undergo a disorder-to-order transition, we also used the ANCHOR algorithm to identify potential binding sites within the disordered regions. As expected, the tandem sequence also presented the highest score within the full protein sequence. In accordance with these findings, our 3D molecular modeling and dynamic simulations of the PvMSP9 structure also indicated the tandem repetitive region as the most disordered, charged and predictably exposed at the surface of merozoites, supporting the idea of this region as critical for protein-protein interactions. The combination of prediction algorithms used for the *In silico* analyses of PvMSP9 were especially interesting given that the mechanism used by MSP9 to be located at the parasite membrane, which is not through a GPI anchor, and its role in merozoite invasion remain unknown [64]. Based on the evidence that specific antibodies against PvMSP9 are able to inhibit the parasite invasion [28], we could hypothesize that antibodies against the repeat regions could have functional activity by inhibiting the formation of MSPs at the surface of merozoites or modify the kinetics of merozoite invasion. In summary, based on the combination of an elevated predicted score in linear B-cell epitope prediction and the highest probability of being inserted in an IUR and located in a binding region of PvMSP9, the sequence EAAPENAEPVHENA designated as PvMSP9_E795-A808_ was selected for validation as a linear B-cell epitope.

In a cross-sectional study carried out using plasma samples from naturally exposed individuals we firstly confirmed the previously described role of two blocks of tandem repeats at the C-terminal region of PvMSP9 (PvMSP9-RIRII) as target of immune response [26, 31]. The high frequency of responders to PvMSP9-RIRII and the RIs were also consistent with previous studies, which describe the two blocks of repeats as the most immunogenic region of PvMSP9 in adults from the Brazilian Amazon [26]. Among the responders against the recombinant protein PvMSP9-RIRII, the majority of individuals were also reactive to the synthetic peptide representing the predicted epitope PvMSP9_E795-A808_, confirming that is naturally immunogenic and supporting the *in silico* prediction workflow used. Interestingly, a significant proportion of responders to PvMSP9-RIRII presented no reactivity against the synthetic peptide. Taking into account that the linear epitope is located in the first block of repeats and the RIs of IgG against PvMSP9-RIRII were higher in individuals who were also responders to PvMSP9_E795-A808_, we believe that non-responders could have had their antibody responses biased towards the second block of repeats, which was also reported as highly immunogenic in our earlier studies [26]. Indeed, the lack of a significant linear epitope predicted in the second block of repeats could suggest that humoral immune responses detected in our previous studies could be directed to conformational epitopes presented in the recombinant PvMSP9-RII. In fact, the lack of peptide-based methods well-stablished for screening these conformational epitopes that we hypothesize limited our findings. On the other hand, the observation of higher antibody levels against PvMSP9-RIRII than PvMSP9-RII only in responders to the peptide and, even with a lower coefficient, the positive correlation between reactivity indexes of IgG antibodies specific to PvMSP9_E795-A808_ and PvMSP9-RIRII, suggested that PvMSP9_E795-A808_ is a linear and naturally immunogenic epitope with significant effect on the humoral immune response directed against the first block of tandem repeats of PvMSP9.

The importance of a linear B-cell epitope within a vaccine candidate against *P*. *vivax* was also described using a similar approach to study PvAMA-1 [57]. However, even though the higher RI found in comparison with our PvMSP9 derived peptide, there was no association between the high response observed and exposure and/or protection, as well as the subclass profiling of responders. In our work, we found a prevalence of cytophilic IgG1 antibodies that were both reactive and non-reactive to PvMSP9_E795-A808_. The high prevalence of cytophilic antibodies to PvMSP9_E795-A808_ was an encouraging finding based on the reported function of such antibodies in the protective immune response to merozoite antigens [65, 66]. In the context of blood-stage malaria immunity against *P*. *falciparum*, the interaction of cytophilic antibodies (IgG1 and IgG3) with monocytes has been extensively reported as important to mediate the effective antibody-dependent cellular inhibition (ADCI) [67–70]. Conversely, the non-cytophilic responses could interfere with the opsonizing effects of IgG1 and IgG3 [68, 71]. Although the effector mechanism of opsonizing antibodies remains controversial in *P*. *vivax* malaria, IgG1 and IgG3 subclasses seem to be important. For examples, IgG1 and IgG3 specific to PvMSP1 were the most prevalent IgG subclasses in asymptomatic individuals from Papua New Guinea [72] and from Brazil, respectively [72–74], suggesting a protective role of these antibodies. Curiously, no correlations were observed between exposure or indicative of protection data and IgG subclass reactivity against PvMSP9-RIRII [26]. In this scenario, the prevalence of IgG1 against PvMSP9_E795-A808_ and the positive ratio between cytophilic and non-cytophilic antibodies (data not show) against the selected peptide suggest the potential for this epitope in immunity acquisition.

Lastly, taking into account that the immune response and susceptibility to malaria are intrinsically linked and vary considerably under different epidemiological scenarios [75], we accessed the relationship between the specific immune response against the linear peptide. We used the years of residence in endemic areas and the self-reported number of malaria lifetime episodes as exposure parameters. Moreover, a crude approximation of protection status estimated by the length of the period (in months) since their last malaria episode and the number of infections within the last 6 months prior to the blood collection. Our first results suggested that antibodies against PvMSP9-RIRII increase with exposure and could be involved in protection, since we observed that responders to PvMSP9-RIRII presented longer time elapsed since the last malaria episode. Additionally, the positive correlation between RIs of IgG specific to recombinant protein and time of residence in the endemic area confirm the cumulative response against the block of tandem repeats in naturally exposed individuals. These findings were corroborated in comparison with previous studies in which a low frequency of antibody responses against PvMSP9-RIRII was reported in children [31] and high frequency in adults [26]. The role of anti-PvMSP9_E795-A808_ IgG antibodies in this process remain unknown, since there are no significant differences in these parameters between responders and non-responders against the peptide, as well as specific correlations between reactivity indexes and exposure and/or protection parameters used. However, among responders to PvMSP9_E795-A808_, the reactivity of IgG antibodies specific to PvMSP9-RIRII presented a direct correlation with time since the last malaria episode and an inverse correlation with the number of malaria episodes in the last six months. Therefore, although our results did not show a clear association between IgG against PvMSP9_E795-A808_ and epidemiological parameters, the direct correlation between IgG1 and the time elapsed since the last malaria episode suggest that the response against PvMSP9_E795-A808_ could be involved in immunity acquisition mediated by naturally acquired antibodies against PvMSP9.

In conclusion, we identified and confirmed that the PvMSP9 peptide sequence EAAPENAEPVHENA (PvMSP9_E795-A808_) contains a linear B-cell epitope. The epitope is present in the protein sequence within a tandem block of repeated amino acids and is targeted by naturally acquired IgG antibodies from individuals living in malaria endemic areas. Antibodies against the linear B-cell epitope were responsible for a significant proportion of immune responses against the entire repetitive region (PvMSP9-RIRII) expressed as a recombinant protein. Lastly, immune responses observed were mainly biased to cytophilic antibodies and the levels of specific IgG1 against the epitope were correlated with epidemiological parameters of protection. Hence, our data describes the potential of PvMSP9_E795-A808_ as an immunogenic linear epitope and support its inclusion in future multi-target vaccine development assays that use synthetic peptides.

## Supporting Information

S1 Fig SDS-PAGE of PvMSP9-RIRII recombinant protein expressed, purified and used as antigen ELISA assays. Lane 1 corresponds to Pre-stained molecular mass markers from BioRad (Precision Plus protein standards, Cat# 161–0373). Lanes 2–3 correspond to rPvMSP9-RIRII in 2.5 μg and 5.0 μg. The recombinant protein migration ≈ 60 Kda confirmed the successfully expression and purity of our antigen, which has a expected molecular mass of 52.1 Kda (25.1 Kda of protein sequence and 27 Kda of GST Tag).
(EPS)
